# Enhancing Surgical Efficiency and Cost‐Effectiveness With TaTME‐Combined Robot‐Assisted Surgery for Lower Rectal Cancer

**DOI:** 10.1002/ags3.70134

**Published:** 2025-11-22

**Authors:** Takeru Matsuda, Hiroshi Hasegawa, Yasunori Otowa, Masayuki Ando, Tomoaki Aoki, Yasufumi Koterazawa, Naoki Urakawa, Hironobu Goto, Shingo Kanaji, Yoshihiro Kakeji

**Affiliations:** ^1^ Division of Gastrointestinal Surgery, Department of Surgery Kobe University Graduate School of Medicine Kobe Japan; ^2^ Division of Minimally Invasive Surgery, Department of Surgery Kobe University Graduate School of Medicine Kobe Japan

**Keywords:** cost, rectal cancer, robotic surgery, TaTME

## Abstract

**Background:**

Robot‐assisted rectal surgery (RAS) offers improved dexterity and visualization; however, the high cost of equipment and consumables remains a major challenge for hospital management. At our institution, we have adopted a combined approach using transanal total mesorectal excision (TaTME) for lower rectal cancers, aiming to shorten operative time and enable two procedures per day to improve cost‐efficiency.

**Methods:**

This retrospective cohort study included 112 patients who underwent RAS for rectal cancer between 2019 and 2024, excluding those requiring lateral lymph node dissection. Patients were divided into a robotic‐only group (Ra/RS tumors, *n* = 75) and a TaTME‐combined group (Rb tumors, *n* = 37). Surgical outcomes and institutional profitability per case were compared, including the number of cases performed twice daily.

**Results:**

Despite much lower tumor height from the anal verge (3 vs. 10 cm, *p* < 0.001), the TaTME‐combined group showed significantly shorter operative time than the robotic‐only group (235 vs. 317 min, *p* < 0.001). Two‐case‐per‐day robotic operations were achieved in 73.0% of the TaTME‐combined group (27 patients) compared to only 20% in the robotic‐only group (15 patients). Oncological outcomes and complication rates were comparable between the groups. In the TaTME‐combined group, performing two procedures per day resulted in a greater gross profit per case (JPY 321 800/case) compared to performing one conventional robotic LAR per day (JPY 224 300/case).

**Conclusions:**

TaTME‐combined RAS for lower rectal cancer enables efficient surgical scheduling and improved cost‐effectiveness while maintaining oncological safety. This strategy holds promise as a model for economically sustainable rectal cancer care.

## 1 Introduction

Robot‐assisted surgery (RAS) for rectal cancer has rapidly gained acceptance as a minimally invasive approach offering superior precision and stable visualization [1, 2]. In particular, RAS has demonstrated clear advantages in the narrow confines of the deep pelvis, allowing for improved nerve preservation and meticulous dissection compared to conventional laparoscopic techniques [2, 3, 4].

However, RAS is not without its limitations. First, the substantial cost of robotic systems, including capital investment, maintenance, and consumables, imposes a significant financial burden on medical institutions [5, 6, 7]. Second, the operative time for RAS tends to be longer than that of conventional surgery due to factors such as docking, setup, and instrument exchange, which affects the efficiency of operating room utilization and overall throughput [8, 9].

To address these limitations, our institution has introduced a combined strategy for lower rectal cancers (Rb tumors) utilizing transanal total mesorectal excision (TaTME) in conjunction with RAS [10]. TaTME allows for direct visualization and precise dissection from the anal side, facilitating faster and safer mobilization of the distal rectum [11, 12]. By integrating TaTME into the robotic workflow, we aim to reduce operative time and enable two‐case‐per‐day scheduling, thereby improving both cost‐efficiency and resource utilization.

This study aims to evaluate the clinical and economic utility of TaTME‐combined RAS for lower rectal cancer, compared to robotic surgery alone for more proximal tumors (Ra and RS), focusing on operative time, postoperative outcomes, and per‐case profitability.

## 2 Materials and Methods

### 2.1 Patients and Study Design

This was a retrospective, single‐center cohort study of patients who underwent robot‐assisted rectal cancer surgery between January 2019 and March 2024. The inclusion criteria are patients with rectal malignant tumor who received RAS. The exclusion criteria are patients who required lateral lymph node dissection, total pelvic exenteration or para‐aortic lymph node dissection. In our institution, as a rule, patients with middle and upper rectal cancers (Ra and RS tumors) are treated with the robot alone, while lower rectal cancers (Rb tumors) are operated with TaTME‐combined approach. Thus, based on tumor location, patients were categorized into two groups: the robotic‐only group (Ra/RS tumors) and the TaTME‐combined group (Rb tumors), in which TaTME was performed in conjunction with robotic surgery. However, this allocation was not strictly enforced. In certain cases, patients with Rb tumors received robotic‐only surgery due to limited availability of TaTME‐trained personnel. Conversely, some patients with bulky Ra tumors underwent TaTME‐combined surgery based on a multidisciplinary assessment of technical feasibility and oncologic safety. All sphincter‐preserving procedures involving the TaTME‐combined approach are routinely accompanied by a diverting ileostomy, regardless of individual risk factors, as part of our standardized safety protocol. In contrast, in the robotic‐only group, a diverting stoma was selectively constructed in patients who had received neoadjuvant therapy. Tumors were categorized based on the Japanese Classification of Colorectal, Appendiceal, and Anal Carcinoma [13].

This study was approved by the institutional review board of our hospital (Approval No. B250113).

### 2.2 Definition

In this study, a “dual‐case day” was defined as a single operating day during which two robot‐assisted rectal resections were scheduled and performed in the same robotic operating theatre. At our institution, two consecutive robotic procedures are routinely accommodated within a standard 8‐h operating room block (approximately 4 h per case).

Although some procedures occasionally exceeded the target time, any day on which two robotic rectal surgeries were carried out was classified as a valid “dual‐case” instance, regardless of whether the combined operative duration slightly exceeded 8 h.

This definition reflects the institutional scheduling policy and resource allocation model rather than strict time‐based completion criteria.

### 2.3 Outcome Measurement

Surgical outcomes were compared between the two groups, including operative time, estimated blood loss, postoperative complications (Clavien‐Dindo grade II or higher), length of hospital stay, pathological outcomes and resection margin status.

To assess economic performance, the approximate profit per case was calculated by subtracting estimated costs—such as depreciation of the robotic system and consumables—from surgical reimbursement under the Japanese health insurance system. Additionally, the proportion of cases performed as two robotic procedures in a single day (dual‐case strategy) was recorded for each group. The assessment of the two‐case‐per‐day strategy was based on the total number of robotic surgeries performed, including procedures other than rectal cancer.

All costs are based on publicly available list prices (rounded down to the nearest thousand yen), and do not include personnel or facility costs. Although personnel costs are typically considered in cost analyses, they were excluded in this study based on the institutional context in Japan. Specifically, additional salary costs are only incurred for overtime work, whereas procedures completed within standard working hours do not increase staffing expenses. Therefore, under the assumption that all procedures were completed during regular working hours, personnel costs were not included in the cost calculations.

In both groups, the AirSeal system (CONMED, NY, USA) was utilized, but in different phases of the surgery. In the robotic‐only group, AirSeal was employed during the robotic phase for stable pneumoperitoneum and smoke evacuation. In the TaTME‐combined group, the AirSeal system was used exclusively during the transanal phase, while the robotic phase utilized a conventional CO_2_ insufflator and standard smoke evacuation system. This phase‐specific usage was reflected in the calculation of consumable costs.

### 2.4 Statistical Analysis

Continuous variables were analyzed using the Student's *t*‐test or Mann–Whitney *U* test, and categorical variables using the chi‐square test or Fisher's exact test, as appropriate. A *P*‐value of < 0.05 was considered statistically significant. All statistical analyses were conducted using EZR (Saitama Medical Center, Jichi Medical University, Saitama, Japan), a graphical user interface for R.

## 3 Results

### 3.1 Baseline Characteristics

Between January 2019 and March 2024, 75 patients with middle or upper rectal tumors (RS or Ra tumors) received the RAS alone, while 61 patients with low rectal tumors (Rb tumors) received TaTME‐combined RAS. Among 61 patients with TaTME‐combined RAS, 3 with TPE, 20 with LLND, and 1 with para‐aortic lymph node dissection were excluded. Finally, 75 patients in the robotic‐only group and 37 patients in the TaTME‐combined group were subjected to the final analysis. Baseline characteristics are shown in Table 1. The TaTME‐combined group included a higher proportion of patients with tumors located in the lower rectum (tumor distance from anal verge: 3.0 vs. 10 cm, *p* < 0.001) and had a greater incidence of neoadjuvant and adjuvant treatment compared to the robotic‐only group.

**TABLE 1:** Baseline characteristics.

	Robotic‐only *n* = 75	TaTME‐combined *n* = 37	*p*
Age, median (range)	70 (41–87)	62 (37–86)	0.054
Sex, *n* (%)			0.535
Male	45 (60.0)	25 (67.6)	
Female	30 (40.0)	12 (32.4)	
BMI (kg/m^2^), median (range)	22.8 (16.2–35.8)	22.0 (14.0–28.0)	0.514
ASA score, *n* (%)			0.137
I	10 (13.3)	4 (10.8)	
II	52 (69.3)	30 (81.1)	
III	13 (17.3)	2 (5.4)	
IV	0 (0)	1 (2.7)	
Neoadjuvant therapy, *n* (%)			< 0.001
Yes	5 (6.7)	13 (35.1)	
No	70 (93.3)	24 (64.9)	
Tumor distance from AV (cm), median (range)	10.0 (3.0–20.0)	3.0 (0–8.0)	< 0.001
Main tumor location^a^, *n* (%)			
RS	19 (25.3)	0 (0)	
Ra	43 (57.3)	3 (8.1)	
Rb	13 (17.3)	27 (73.0)	
P	0 (0)	7 (18.9)	
cT^a^, *n* (%)			0.884
1	18 (24.0)	9 (24.3)	
2	17 (22.7)	9 (24.3)	
3	28 (37.3)	14 (37.8)	
4a	18 (24.0)	4 (10.8)	
4b	4 (5.3)	1 (2.7)	
cN^a^, *n* (%)			0.940
0	44 (58.7)	24 (64.9)	
1	20 (26.7)	9 (24.3)	
2	9 (12.0)	3 (8.1)	
3	2 (2.7)	1 (2.7)	
cStage^a^, *n* (%)			0.582
I	32 (42.7)	17 (45.9)	
II	10 (13.3)	7 (18.9)	
III	27 (36.0)	9 (24.3)	
IV	6 (8.0)	4 (10.8)	
Adjuvant chemotherapy, *n* (%)			0.035
Yes	14 (18.9)	14 (38.9)	
No	60 (81.1)	22 (61.1)	

Abbreviations: AV, anal verge; BMI, body mass index; iASA, American Society of Anesthesiologists.

^a^ According to the Japanese Classification of Colorectal, Appendiceal, and Anal carcinoma.

### 3.2 Operative and Postoperative Outcomes

The operative and postoperative outcomes are shown in Table 2. In the TaTME‐combined group, intersphincteric resection (ISR) and abdominoperineal resection were performed in 4 (10.8%) and 13 patients (35.1%), respectively, while none received those surgeries in the robotic‐only group. The median operative time was significantly shorter in the TaTME‐combined group compared to the robotic‐only group (235 vs. 317 min, *p* < 0.001).

**TABLE 2:** Operative and postoperative outcomes.

	Robotic‐only *n = 75*	TaTME‐combined *n = 37*	*P*
Operative procedure, *n* (%)			< 0.001
High anterior resection	14 (18.7)	0	
Low anterior resection	57 (76.0)	20 (54.1)	
Intersphincteric resection	0	4 (10.8)	
Abdominoperineal resection	0	13 (35.1)	
Hartmann procedure	4 (5.3)	0	
Diverting ileostomy, *n* (%)	3 (4.0)	24 (64.9)	< 0.001
Operation time^a^, min (range)	317 (215–577)	235 (167–385)	< 0.001
Estimated blood loss^a^, g (range)	0 (0–200)	0 (0–200)	0.120
Transfusion, *n* (%)	1 (1.3)	2 (5.4)	0.253
Conversion to open surgery, *n* (%)	0	1 (2.7)	0.330
Postoperative complications (CD ≥ II), *n* (%)	19 (25.3)	10 (27.0)	1
Urinary disturbance	5 (6.7)	0	
Anastomotic leakage	4 (5.3)	3 (8.1)	
Perineal wound infection	0	2 (5.4)	
Abdominal abscess	3 (4.0)	3 (8.1)	
Bowel obstruction	3 (4.0)	0	
Paralytic ileus	2 (2.7)	0	
Lymphorrhea	1 (1.3)	0	
Pneumonia	1 (1.3)	0	
Others	4 (5.3)	2 (5.4)	
Postoperative complications (CD ≥ III), *n* (%)	6 (8.0)	3 (8.1)	1
Postoperative hospital stay (day)^a^, median (range)	12 (9–90)	18 (9–70)	< 0.001
Mortality within 30 days, *n* (%)	0	0	1
Cases completed within a two‐case‐per‐day schedule^b^, *n* (%)	15 (20.0)	27 (73.0)	< 0.001

Abbreviation: CD, Clavien‐Dindo classification.

^a^ The data are expressed as the median (range).

^b^ This variable indicates the number of procedures completed as the first or second case of a two‐case‐per‐day robotic surgical schedule.

No significant differences were observed in postoperative complication rates (Clavien‐Dindo grade II or higher: 25.3% vs. 27.0%, *p* = 1), while postoperative hospital stay was significantly longer in the TaTME‐combined group (12 vs. 18 days, *p* < 0.001).

### 3.3 Pathological Outcomes

The pathological outcomes are summarized in Table 3. There were significant differences only in the histological type. The positive circumferential resection margin (CRM) was observed in 1 (1.3%) of the robotic‐only and 2 (5.4%) of the TaTME‐combined groups, respectively (*p* = 0.104).

**TABLE 3:** Pathological outcomes.

	Robotic‐only *n* = 75	TaTME‐combined *n* = 37	*p*
Histological type, *n* (%)			0.021
Differentiated	71 (94.7)	26 (72.2)	
Undifferentiated	1 (1.3)	3 (8.3)	
Other	3 (4.0)	7 (19.4)	
(y)pT^a^, *n* (%)			0.741
0/is	2 (2.7)	3 (8.1)	
1	20 (26.7)	9 (24.3)	
2	13 (17.3)	10 (27.0)	
3	37 (49.3)	15 (40.5)	
4	3 (4.0)	0	
(y)pN^a^, *n* (%)			0.224
0	51 (68.0)	21 (56.8)	
1	13 (17.3)	12 (32.4)	
2	11 (14.7)	4 (10.8)	
3	0	0	
(y)pStage^a^, *n* (%)			0.570
0	1 (1.3)	2 (5.4)	
I	28 (37.3)	13 (35.1)	
II	19 (25.3)	6 (16.2)	
III	20 (26.7)	12 (32.4)	
IV	7 (9.3)	4 (10.8)	
Lymphatic invasion, *n* (%)			0.218
Negative	25 (33.3)	17 (45.9)	
Positive	50 (66.7)	20 (54.1)	
Vascular invasion, *n* (%)			0.422
Negative	35 (46.7)	21 (56.8)	
Positive	40 (53.3)	16 (43.2)	
DM involvement, *n* (%)	0 (0)	0 (0)	1
CRM involvement, *n* (%)	1 (1.3)	2 (5.4)	0.104

Abbreviations: DM, distal margin; iCRM, circumferential resection margin.

^a^ According to the Japanese Classification of Colorectal, Appendiceal, and Anal carcinoma.

### 3.4 Efficiency and Cost Analysis

The proportion of cases performed as two robotic procedures in one day was significantly higher in the TaTME group (27 patients, 73.0%) compared to the robotic‐only group (15 patients, 20.0%, *p* < 0.001) (Table 2). Table 4 summarizes the estimated gross profits for the TaTME‐combined and robotic‐only groups under two different scenarios: performing one case per day versus two cases per day. In the TaTME‐combined group, performing two robotic procedures per day resulted in a substantially greater gross profit per case (+321 800 JPY) compared to performing one conventional robotic LAR per day (+224 300 JPY).

**TABLE 4:** Cost comparison of robotic rectal surgery approaches.

Category	Item	Unit cost (JPY)	Quantity	Subtotal (JPY)
*Robotic LAR (1 case/day)*				
Robot	Depreciation & Maintenance (7 years/250 days usage)	210 000	1	210 000
Instruments	Fenestrated bipolar	29 000	1	29 000
Instruments	Monopolar curved scissors	41 000	1	41 000
Instruments	Tip‐up forceps	33 000	1	33 000
Energy device	Vessel‐sealing device	90 000	1	90 000
Stapler	Linear stapler	70 000	1	70 000
Stapler	Circular stapler	80 000	1	80 000
AirSeal system	Trocar and filter tube	52 000	1	52 000
Disposable trocar	Trocar	10 000	1	10 000
Total (JPY)				615 000
Gross profit (JPY)^a^				+224 300
*Robotic LAR (2 cases/day)*				
Robot	Depreciation & Maintenance (7 years/250 days usage)	105 000	1	105 000
Instruments	Fenestrated bipolar	29 000	1	29 000
Instruments	Monopolar curved scissors	41 000	1	41 000
Instruments	Tip‐up forceps	33 000	1	33 000
Energy device	Vessel‐sealing device	90 000	1	90 000
Stapler	Linear stapler	70 000	1	70 000
Stapler	Circular stapler	80 000	1	80 000
AirSeal system	Trocar and filter tube	52 000	1	52 000
Disposable trocar	Trocar	10 000	1	10 000
Total (JPY)				510 000
Gross profit (JPY)^a^				+329 300
*TaTME‐combined robotic LAR (1 case/day)*				
Robot	Depreciation & Maintenance (7 years/250 days usage)	210 000	1	210 000
Instruments	Fenestrated bipolar	29 000	1	29 000
Instruments	Monopolar curved scissors	41 000	1	41 000
Instruments	Tip‐up forceps	33 000	1	33 000
Energy device	Vessel‐sealing device	90 000	1	90 000
Stapler	Linear stapler	70 000	0	0
Stapler	Circular stapler	80 000	1	80 000
Disposable trocar	Trocar	10 000	1	10 000
TaTME device	GelPOINT Path	70 000	1	70 000
Laparoscopic Equipment	Depreciation & Maintenance (7 years/250 days usage)	15 000	1	15 000
AirSeal system	Trocar and filter tube	52 000	1	52 000
Total (JPY)				630 000
Gross profit (JPY)^a^				+209 300
*TaTME‐combined robotic LAR (2 cases/day)*				
Robot	Depreciation & Maintenance (7 years/250 days usage)	105 000	1	105 000
Instruments	Fenestrated bipolar	29 000	1	29 000
Instruments	Monopolar curved scissors	41 000	1	41 000
Instruments	Tip‐up forceps	33 000	1	33 000
Energy device	Vessel‐sealing device	90 000	1	90 000
Stapler	Linear stapler	70 000	0	0
Stapler	Circular stapler	80 000	1	80 000
Disposable trocar	Trocar	10 000	1	10 000
TaTME device	GelPOINT Path	70 000	1	70 000
Laparoscopic Equipment	Depreciation & Maintenance (7 years/250 days usage)	7500	1	7500
AirSeal system	Trocar and filter tube	52 000	1	52 000
Total (JPY)				517 500
Gross profit (JPY)^a^				+321 800

*Note:* All listed prices are approximate and rounded down to the nearest thousand JPY (Japanese Yen) based on official list prices.

^a^ The gross profit was calculated by subtracting the total estimated cost from the standard operation reimbursement fee of 839 300 JPY for standard low anterior resection under the Japanese National Health Insurance system.

To assess the impact of overtime on personnel cost, we analyzed surgical end‐times and estimated overtime‐related surgeon wages based on the number of surgeons involved per procedure. As shown in Table 5, the TaTME‐combined group demonstrated a higher proportion of cases completed within regular working hours and incurred a lower total estimated overtime cost, despite involving more surgeons per procedure.

**TABLE 5:** Summary of regular‐hour completion rates and overtime workload.

	Robotic‐only *n = 75*	TaTME‐combined *n = 37*
Cases completed within regular hours, *n* (%)	64 (85.3)	34 (91.9)
Cases with overtime, *n* (%)	11 (14.7)	3 (8.1)
Median overtime, min (range)	61 (10–154)	30 (12–65)
Estimated additional cost per case (JPY)^a^	20 333	10 000

*Note:* Overtime cost was estimated only for surgeons, with an hourly wage of JPY10 000 per person. The robotic‐only group was staffed with 2 surgeons, and the TaTME‐combined group with 4 surgeons, reflecting institutional practice. Nurses and medical engineers were not included in this estimation, as their duties beyond regular hours are typically absorbed by pre‐scheduled evening shift personnel without extra wage outlay.

^a^ Data are expressed as median.

## 4 Discussion

This study demonstrated that the combination of TaTME with RAS for lower rectal cancer significantly shortened operative time compared to RAS alone for upper or mid rectal cancer. Moreover, the TaTME‐combined group enabled a greater proportion of two‐case‐per‐day procedures, resulting in superior operating room efficiency and improved institutional profitability. These findings suggest that the TaTME‐assisted approach can be a practical solution to the economic and logistical limitations commonly associated with robotic surgery.

Although RAS offers clear advantages in pelvic surgery, such as enhanced dexterity and visualization, it also presents inherent disadvantages, including longer operative times and increased costs due to robotic set‐up, docking, and specialized instruments. The integration of TaTME addresses these challenges by allowing efficient and direct dissection of the distal rectum from the anal side, which is often the most time‐consuming part of the procedure. In our cohort, TaTME‐combined RAS significantly reduced operative time by over 60 min without compromising oncologic outcomes despite of lower rectal cancers.

Importantly, the oncologic safety of TaTME‐combined RAS for lower rectal cancer was comparable to that of robotic surgery alone for upper or mid rectal cancer, although previous reports from Norway and the Netherlands have raised concerns regarding the oncological safety of TaTME [14, 15]. No significant differences were observed between the groups in terms of postoperative complications and positive CRM rates. These findings affirm that surgical efficiency can be achieved without compromising quality.

Furthermore, the enhanced operability of TaTME allowed for more consistent scheduling of two robotic cases per day, increasing the effective use of the robotic platform. Financial analysis revealed that the profit per case was substantially higher in the TaTME group, underscoring the cost‐effectiveness of this combined approach under current reimbursement structures. If robotic‐only surgery were to be applied to lower rectal cancer cases, the operative time would likely be further prolonged, making the two‐case‐per‐day strategy even more challenging to implement. In addition, efficient patient turnover is a critical component in implementing the dual‐case strategy. Although no formal turnover protocols or external task forces were employed, the surgical team—including attending surgeons—actively participated in room cleaning and patient transfer between cases. This collaborative approach facilitated efficient transitions and contributed to the feasibility of completing two robotic rectal surgeries within standard operating hours.

Another important consideration is the difference in diverting stoma rates between the two groups. In our institutional protocol, all sphincter‐preserving procedures involving the TaTME‐combined approach are accompanied by a diverting ileostomy, irrespective of individual risk factors such as anastomotic height, neoadjuvant chemoradiotherapy, or patient background. This policy was implemented as a standardized safety measure to minimize the clinical impact of anastomotic leakage in technically demanding low pelvic anastomoses. In contrast, in the robotic‐only group, diverting stomas were selectively constructed only for patients who had undergone neoadjuvant chemoradiotherapy. We acknowledge that this policy inevitably led to a higher stoma rate in the TaTME‐combined group and thus an additional patient burden in terms of postoperative quality of life and resource use. However, this difference reflects an institutional safety strategy rather than a technical limitation of the TaTME procedure itself. Future studies evaluating patient‐reported outcomes and cost implications after stoma reversal will be important to fully assess the overall balance between safety, efficiency, and quality of life in each surgical approach.

Although this analysis used the reimbursement fee for a standard LAR (839 300JPY) as the basis for cost evaluation, it should be noted that the majority of TaTME‐combined cases in this study involved lower rectal tumors, often requiring ISR or ultra‐low anterior resection with transanal anastomosis. As these procedures generally yield higher reimbursement, the actual cost‐effectiveness of the TaTME‐combined approach is likely to be even greater than estimated.

The reproducibility of TaTME‐combined robotic surgery in general hospitals may be limited by the availability of experienced surgeons, dedicated multidisciplinary teams, and dual‐console systems. However, as structured training programs and proctoring systems have been introduced in recent years, safe and standardized implementation of TaTME has become increasingly feasible outside high‐volume centers. Additionally, the use of two parallel teams (transabdominal and transanal) enables a more efficient use of time and resources, which may be especially valuable in institutions facing limited operating room availability. Our results could serve as a benchmark model for centers aiming to optimize resource utilization while maintaining surgical quality.

Several limitations must be acknowledged. This was a single‐institution, retrospective study with a limited sample size. Moreover, the institutional strategy to select robotic‐only or TaTME‐combined procedures based on tumor location (i.e., upper rectum and rectosigmoid lesions for robotic‐only, lower rectum and anal canal for TaTME‐combined) precluded statistical background adjustment or matching between groups. Therefore, readers are advised to interpret the between‐group comparisons as exploratory rather than causally inferential. Future multi‐institutional and prospective studies are warranted to validate the generalizability and long‐term outcomes of this strategy.

In conclusion, TaTME‐combined RAS for lower rectal cancer achieved significant reductions in operative time and enabled a two‐case‐per‐day strategy, addressing both time and cost inefficiencies inherent to robotic surgery. This approach maintained oncologic safety while improving institutional profitability, making it a valuable model for sustainable and high‐quality rectal cancer care in the robotic era.

## Author Contributions

**Takeru Matsuda:** conceptualization, investigation, writing – original draft, formal analysis, methodology. **Hiroshi Hasegawa:** methodology, writing – review and editing, formal analysis. **Yasunori Otowa:** methodology, formal analysis, writing – review and editing, data curation. **Masayuki Ando:** methodology, writing – review and editing, formal analysis, data curation. **Tomoaki Aoki:** writing – review and editing, formal analysis, data curation. **Yasufumi Koterazawa:** writing – review and editing, formal analysis, data curation. **Naoki Urakawa:** writing – review and editing, investigation, formal analysis. **Hironobu Goto:** investigation, writing – review and editing, formal analysis. **Shingo Kanaji:** supervision, writing – review and editing, project administration. **Yoshihiro Kakeji:** writing – review and editing, project administration, supervision.

## Funding

The authors have nothing to report.

## Ethics Statement

Approval was obtained from the Institutional Review Board of Kobe University Hospital (IRB number: B250113). The study was conducted in accordance with Good Clinical Practice guidelines and the Declaration of Helsinki.

## Conflicts of Interest

Takeru Matsuda and Yoshihiro Kakeji are Editorial Board Members of *Annals of Gastroenterological Surgery*.

## Data Availability

The data that support the findings of this study are available from the corresponding author upon reasonable request.
